# Developing clinical practice guidelines: target audiences, identifying topics for guidelines, guideline group composition and functioning and conflicts of interest

**DOI:** 10.1186/1748-5908-7-60

**Published:** 2012-07-04

**Authors:** Martin P Eccles, Jeremy M Grimshaw, Paul Shekelle, Holger J Schünemann, Steven Woolf

**Affiliations:** 1 Institute of Health and Society, Newcastle University, Baddiley-Clark Building, Richardson Road, Newcastle upon Tyne, NE2 4AX, UK; 2 Clinical Epidemiology Program, Ottawa Hospital Research Institute, Ottawa, ON, Canada; 3 Department of Medicine, University of Ottawa, Ottawa, ON, Canada; 4 RAND Corporation, Santa Monica, CA, 90407, USA; 5 Veterans Affairs Greater Los Angeles Healthcare System, Los Angeles, CA, 90073, USA; 6 Departments of Clinical Epidemiology and Biostatistics and of Medicine, McMaster University, Hamilton, Canada; 7 Department of Family Medicine and Center on Human Needs, Virginia Commonwealth University, Richmond, VA, USA

## Abstract

Clinical practice guidelines are one of the foundations of efforts to improve health care. In 1999, we authored a paper about methods to develop guidelines. Since it was published, the methods of guideline development have progressed both in terms of methods and necessary procedures and the context for guideline development has changed with the emergence of guideline clearing houses and large scale guideline production organisations (such as the UK National Institute for Health and Clinical Excellence). It therefore seems timely to, in a series of three articles, update and extend our earlier paper. In this first paper we discuss: the target audience(s) for guidelines and their use of guidelines; identifying topics for guidelines; guideline group composition (including consumer involvement) and the processes by which guideline groups function and the important procedural issue of managing conflicts of interest in guideline development.

## Background

Clinical practice guidelines (hereafter referred to as guidelines) are one of the foundations of efforts to improve health care. The modern age of guidelines began with a 1992. Institute of Medicine report, which defined guidelines as “systematically developed statements to assist practitioner and patient decisions about appropriate health care forspecific clinical circumstances”. [1] In 1999, we authored a paper about methods to develop guidelines. [2] It covered: identifying and refining the subject area of the guideline; running guideline development groups; identifying and assessing the evidence; translating evidence into a clinical practice guideline; and reviewing and updating guidelines. Since it was published, the methods of guideline development have progressed both in terms of methods and necessary procedures and the broad context for clinical practice guidelines has changed.

To help users identify and choose guidelines there has been the emergence of guideline clearing houses (such as the AHRQ Guideline Clearing House ( http://www.guideline.gov)) that identify and systematically characterize guidelines on a number of domains and the development of robust guideline appraisal instruments such as the AGREE tool [3,4] There has been the appearance of large scale guideline production organisations both at a national level (such as the UK National Institute for Health and Clinical Excellence or. Scottish Intercollegiate Guidelines Network) and a condition level (such as the Ontario Cancer Guideline Program). There have also been relevant reports (that some of us have participated in) for the World Health Organisation [5]and professional societies (Schünemann HJ, Woodhead M, Anzueto A, Buist AS, MacNee W, Rabe KF, Heffner J. A guide for guidelines for professional societies and other developers of recommendations: an official American Thoracic Society (ATS)/European Respiratory Society (ERS) Workshop Report; forthcoming). Such organizations and those interested in producing and using guidelines now have a high profile society in the Guidelines. International Network ( http://www.g-i-n.net/). Against this background it seems timely to, in a series of three articles, update and extend our earlier paper on the methods of developing clinical practice guidelines. This series is based on a background paper [6] prepared for the Institute of Medicine report “Clinical Practice Guidelines We Can Trust” [7].

In this first paper we discuss: the target audience(s) for guidelines and their use of guidelines; identifying topics for guidelines; guideline group composition (including consumer involvement) and the processes by which guideline groups function and the important procedural issue of managing conflicts of interest in guideline development. Inthe second paper we will move on to discuss issues of identifying and synthesizing evidence: deciding what type of evidence and outcomes to include in guidelines; integrating values into a guideline; incorporating economic considerations; synthesis, grading, presentation of evidence; and moving from evidence to recommendations.Finally, in the third paper we will discuss the issues of: reviewing, reporting, and publishing guidelines; updating guidelines; and the two emerging issues of enhancing guideline implementability and how guideline developers should approach dealing with the issue of patients who will be the subject of guidelines having co-morbid conditions. By updating our previous work we hope to offer to a general reader a useful current overview of the main methodological and procedural issues in guideline development.

### Target audiences and their use of guidelines

An important first step in developing a guideline is to clarify the target audience. A clear sense of the target audience informs subsequent decisions about the guideline’s scope, objectives, and format and style of wording. Typically, guidelines have both *primary* and *secondary* audiences. The primary audience is the category of clinicians (and patients) for whom the guideline is nominally intended and who are most likely to use the guideline in patient care settings. However, guideline developers often recognize a secondary audience that takes considerable interest in the recommendations. For example, a family medicine or paediatrics society may develop guidelines for its practitioners, knowing that other primary care professionals could refer to the guidelines in managing the same condition. Guideline developers are also aware that their recommendations may be used in policy processes such as informing coverage decisions by healthcare providers, insurers and employers’ benefits packages.

Guidelines often inadvertently focus on physicians as the target audience and use the term “physician” or “doctor” in lieu of “clinician,” but the topics they address may be equally relevant to a wider range of clinicians. For example, a guideline on a primary care chronic disease management topic (such as diabetes mellitus) will be relevant not only to primary care physicians but also to nurse practitioners, physician assistants, and other clinicians or social workers who work in primary care settings. Even when the target audience is clearly physicians, it is useful to clarify the type of physician(s) for whom the clinical practice guideline is primarily intended. Guidelines intended for primary care physicians may include content of less interest to specialists, and vice versa. Guidelines on a highly specialized procedure, performed only by sub-specialists, are unlikely to be used by primary care physicians and therefore need not review basic background on the health condition, can focus on narrow evidence questions, and can use specialized terminology without extensive elaboration.

### Prioritizing topics for guideline development

There are many factors that influence the choice of topics for the development of guidelines including scientific criteria set alongside the needs of health care policymakers, specialty society professional identification, and in response to popular media coverage of certain high profile cases. Guidelines can be developed around conditions (diabetes, hypertension, HIV infection, etc.) or around procedures (coronary angiography, colonoscopy, carotid endarterectomy, etc.) The choice has to do with the target audience and the need. Most guidelines are developed for conditions. Among the scientific factors are the criteria in Table 1.

**Table 1:** Criteria for developing guidelines


·	High prevalence condition or high use medical procedure
·	High associated cost
·	Effects on premature mortality and avoidable morbidity
·	Evidence that medical care can make a difference in outcomes
·	Knowledge of current variations in practice, or that practice does not match some known practice parameters

Thus, situations for the development of guidelines are often considered to be those conditions which are common, costly, have large effects on premature mortality or morbidity, for which there is good evidence that appropriate health care can make a difference in outcomes, and for which we know that there are wide variations in current care (as a proxy for professional uncertainty about how to care for the condition) or prima facie knowledge that current care does not meet some well-accepted standards. Clearly, judgment is needed in applying these criteria. There are some conditions that are rare, but for which receiving appropriate medical care is the difference between a normal health outcome and catastrophe (e.g. phenylketonuria). Such conditions should not be dismissed as a low priority for guidelines based solely on prevalence. Similarly, there are some conditions which have only modest effects on morbidity, but which are so common that the cumulative effects of appropriate management at a population level may be large (e.g. acute pharyngitis). Also, the evidence that differences in clinical management lead to differences in health care outcomes does not necessarily have to be positive - for some conditions, too much health care might produce negative individual health or population outcomes (e.g. treating acute upper respiratory infections with antibiotics may cause patients to have antibiotic associated diarrhea or may increase community antibiotic resistance rates).

Most often the decisions about the relative importance of prevalence, cost, effect of health care, etc., are made implicitly; there is little published about how such decisions are made. The US Preventive Services Task Force (USPSTF) has a Topic Prioritization Workgroup that regularly assesses nominations for guidelines that come from professional societies and other groups. [8] The USPSTF does not use a formal scoring system, but rather assesses each topic as a whole, using criteria similar to that presented above, as do NICE and SIGN in the UK. The American College of Physicians Clinical Guidelines Group likewise uses a global judgment method for prioritizing topics [9].

### Guideline group composition and group process

Guideline development involves both a technical process (systematic reviews of relevant evidence) and a social process (interpretation of the results of the systematic review and development of recommendations). The validity of guideline recommendations can be adversely influenced if either process is biased. While there has been considerable methodological focus on ensuring the validity of the technical process, less attention has been paid to optimizing the social process. [10-14] The quality of the social process is dependent upon the composition of the group (whether the right people have been brought around the table) and the group process (whether the process allows all to participate in a constructive discussion around the implications of the evidence).

### Group composition

There is consistent empirical evidence that the composition of recommendation making groups influence the resulting recommendations. Hutchings and colleagues completed a systematic review of factors affecting judgments produced by formal consensus development methods. [15] They identified 22 studies that examined the impact of specialty or profession of individual participants and observed that participants that performed a procedure were likely to rate *more* appropriate indications for that procedure. They also identified five studies that compared the recommendations made by uni-disciplinary and multi-disciplinary groups; in all studies, recommendations by multidisciplinary groups were more conservative. While there is no gold standard to compare with, these studies are usually interpreted to support the use of multidisciplinary recommendation groups. Lomas identified three reasons in support of this: firstly, the limited information available for guideline development needs to be supplemented by the interpretations of these stakeholders; secondly, legitimate conflicts over values need to be resolved; and thirdly, the successful introduction of a guideline requires that all key disciplines contribute to its development to ensure "ownership" and support. [16] Given these empirical and theoretical arguments, there is a broad international consensus that guideline development groups should be multidisciplinary with representation from key stakeholder groups.

The number of group members and the balance of disciplines should be influenced by the focus of the guidelines. When deciding on the composition of the group, all potential stakeholders should be identified including: health care professionals who are directly involved in the clinical management of patients in different health care settings (for example, primary and secondary care), policy makers who may need to make decisions about resource utilization and patients. There then has to be made a decision about which categories of participant to involve within the guideline group. Guideline developers often have to weigh the desire for wide representation against the need for a cohesive working group. Small groups may not have sufficient experience within their members; larger groups may lack cohesiveness and be difficult to lead. In general, the optimum size for a small group is thought to be between eight and ten people, although groups of larger size have operated effectively.

#### Consumer involvement in clinical practice guideline development

There is an increasing (though still limited) literature on consumer involvement in health decision making in general. A systematic review of patient and public involvement programs (PPIPs) in developing clinical practice guidelines [17] looked at stated reasons for including patients and the public within the process. Of 71 descriptive reports (guideline documents or methods reports, evaluative studies) included in the review 23 reported using PPIPs to “incorporate patients’ values, preferences, knowledge, or perspectives in CPG recommendations”. Other stated reasons were to “improve the implementation of the CPG (7/71), increase the comprehensiveness of the CPG (7/71), promote patients’ or the public’s influence over the CPG development process (7/71), and adapt CPGs to the target population (5/71)”.

These issues are reflected in the empirical work of Boivin et al. [18] who, from 18 interviews with patients, health professionals, and public health experts, identified four discourses about including patient preferences in the process of guideline development. Firstly a Governance discourse (concerned with maximizing public health benefits); secondly an Informed discourse (concerned with maximizing consumer choice via information on benefits and harms); thirdly a Professional Care discourse (concerned with individualizing clinical decisions to individual patients); and a Consumer Advocacy discourse (concerned with optimizing consumers political power within the process). The systematic review by Legare [17] represents the most comprehensive analysis of consumer involvement to date though the literature is descriptive. Of the 71 reports included only 28 were dealing with consumers within the guideline development group itself and only 29 were dealing with consumers involved in crafting recommendations. Other methods of consumer involvement included workshops/meetings/seminars, involvement in systematic reviewing, focus groups, individual interviews and public polls/surveys. Across the reports the authors identified a number of barriers to consumer involvement - the discrepancy between the views of patients and experts, challenges of recruitment, obtaining representative input, consumers lack of familiarity with technical issues and the degree of work/time involved. Against these they set the positive impact of training and support.

From the review it appears that there are still few analytical empirical accounts of attempts to involve consumers in guideline development, and there are no robust evaluations of the effectiveness of different methods. A systematic review of the effectiveness of consumer involvement in health decision making [19] only identified six randomized controlled trials in total and did not find any studies in the context of clinical guideline development (despite searching).

Williamson [20] has proposed three types of patient representatives, depending on the contributions and skills that each can bring: (a) fellow patients who would mainly present their own views, (b) a member of a patient group who presents the organization’s position, and (c) patient advocates who present knowledge of patient views. The North of England Evidence Based Guideline Development Program described their experience of four methods of consumer involvement (three of which were based within the process of guideline development and one of which was conducted to explore the potential of the method for future use) [21]. They included:

• incorporating individual patients in guideline development groups;

• a ‘one off’ meeting with patients;

• a series of workshops with patients;

• incorporating a consumer advocate in guideline development groups

Individual patients who participated in a guideline development group contributed infrequently and had problems with the use of technical language. Although they contributed most in discussions of patient education, their contributions were not subsequently acted on. Within a ‘one off’ meeting with a group of patients, participants again encountered problems with medical terminology and were most interested in sections on patient education and self management. Their understanding of the use of scientific evidence to derive more cost-effective care practices was unclear. The workshop format was relative resource intensive but made it possible to explain the technical elements of guideline development, enabling patients to engage in the process and make relevant suggestions. A patient advocate who served on a group felt confident to speak and was accustomed to discussions with health professionals and to medical terminology.

However, the involvement of lay people in practice guideline development can also be problematic. The North of England experiences and Legare’s review [17] demonstrated the challenges of the lack of consumers’ training and scientific literacy (though this can apply in degree to many other guideline group members). Another challenge with consumer involvement occurs when the representative has a visceral personal experience with the disease or an advocacy role that interferes with the ability to examine the evidence and recommendations dispassionately. Such individuals may have difficulty divorcing their personal narrative or policy agenda from the systematic methods and analytic rules a practice guideline group should follow. As a consequence, the group’s orderly review of the evidence and construction of recommendations can be sidelined by the objections and testimonials of the consumer representative.

Several strategies exist for maximizing the benefits of consumer engagement while avoiding the difficulties. For example, as should occur with selecting any member of a practice guideline group, selection criteria can be applied in choosing a consumer representative with the ability to consider the evidence objectively and make recommendations that depart from preconceived views or self interests. The National Institute for Health and Clinical Excellence in the United Kingdom advocates the involvement of at least two consumers on guideline development groups and provides induction sessions that brief consumers on what to expect as participants.

Another option is to not seat a consumer representative on the guideline group itself, limiting the latter to professionals, but eliciting the perspective of patients and the public as part of a larger stakeholder input exercise. For example, a group may not include a consumer representative but may invite patients or other laypersons to review draft documents or attend a group meeting to share their perspective. Guidelines groups can host an open forum, in which various stakeholder groups, such as patients, payers, manufacturers, and professional associations, are afforded the opportunity to express their perspectives and criticisms, present scientific evidence of relevance to the guideline, or raise concerns about the impact or implementation of proposed recommendations [22]. The advantage of this approach is that it exposes the group’s deliberations to concerns and information that it might otherwise overlook and provides stakeholders a sense of “being heard,” but it leaves the group the freedom to then deliberate privately in processing the comments.

### Guideline group processes

A range of psychosocial factors can influence the progress and content of group meetings. [10] Guideline development groups undergo a socialization process (forming, storming, norming, performing, adjourning). [23] For example, during the first few meetings of a group, much attention may be paid to the development of good interpersonal relations, establishing group aims, developing norms of behavior and exploring explicit and implicit roles. Such group-related issues may have to be addressed before substantial progress can be made on the development of clinical recommendations. Group decision-making essentially involves three phases: o*rientation* (defining the problem), *evaluation* (discussion of decision alternatives), and c*ontrol* (deciding which of the alternatives is to prevail). [24] Ideal conditions for group decision-making are those which enable the views of all parties to be expressed and considered before a recommendation that is acceptable to the majority is reached. [10] Dysfunctional group processes that allow undue influence of minority or majority views may result in the production of invalid or unreliable recommendations. Examples include minority influence, group polarization and groupthink. [10] Multidisciplinary groups are particularly at risk in this regard, since their members vary in professional status, in the nature or depth of their specialist knowledge and in their appreciation of the roles and modus operandi of their professional colleagues.

The risk of such psychosocial biases can be reduced with careful planning and attention to small group processes. For example, it may be appropriate to ensure that guideline development groups receive support to ensure that the group both functions effectively (the group process) and achieves its aims (the group task). [25] It is possible for a single individual to be responsible for supporting both the group process and the group task. If a group is particularly large or if the task is particularly complex, however, such leadership roles may be better divided between two individuals, providing that both they and the group are clear about their differing functions. Technical support is required mainly to identify and synthesize evidence and present this to the group in a form that allows them to make recommendations. This needs a range of skills more likely to be found within researchers than clinicians. During guideline development, the person(s) responsible for technical support should encourage the group to scrutinize the guideline repeatedly to ensure its internal logic and clarity.

### Managing conflicts of interest in guideline development

#### What are conflicts of interest?

An aspect that has gained increasing prominence with the “industrialisation” of guideline development is the need to consider conflicts of interest. Financial, intellectual and other investments in aspects of health care can result in biased judgement about a topic of interest; a concept that is generally called “conflict of interest”. A number of organizations have defined “conflicts of interest” (COI) (Table 2). Grilli and co-workers evaluated 431 practice guidelines developed by specialty societies and found that 67% did not report the type of professionals involved in the guideline development. [26]. Therefore, an assessment of the potential influence of COI would not be possible. A study in the journal Nature of over 200 guidelines listed in the US National Guideline Clearinghouse showed that half of the guidelines reported no information regarding funding sources or financial conflicts of interest of the authors [27].

**Table 2:** Definitions of conflicts of interest


A conflict of interest is a set of circumstances that creates a risk that professional judgment or actions regarding a primary interest will be unduly influenced by a secondary interest. [28] (IoM)
“A divergence between an individual’s private interests and his or her professional obligations such that an independent observer might reasonably question whether the individual’s professional actions or decisions are motivated by personal gain, such as financial, academic advancement, clinical revenue streams or community standing” [29]
“A financial or intellectual relationship that may impact an individual’s ability to approach a scientific question with an open mind” [29].

That COI can misinform healthcare decision makers is widely recognized [30,31]. Omission of consideration for COI can damage an organization’s reputation or require time consuming processes of dealing with perceived influence of COI. Examples include guidelines developed by the World Health Organization, the surviving sepsis campaign and the Infectious Disease Society of America Lyme Disease Guidelines [26,32-39]. Declaration and management of COI is therefore of increasing importance for medical professional societies and other organizations developing practice guidelines [29,40,41]. Financial COI is the best known type of COI and typically a result of direct financial benefit related to topics discussed or products recommended in guidelines. These personal financial interests are not limited to employment, consultancies, paid expert 15 testimony, stock holdings, endowments, patents, royalties, honoraria, and in kind gifts (e.g. travel, accommodation, meals, frequent flier miles). Indirect financial interests are a reality as academic advancement can be related to topics discussed in guidelines. Direct guideline funding by for-profit organizations is another common problem and some authors have requested that professional medical organizations reject all industry funding for practice guidelines [42].

Intellectual COI is another type of conflict [29] that is increasingly recognized and results from a guideline group member being invested in her/her intellectual work. Other examples include authorship of original studies and peer-reviewed grant funding directly related to recommendations under consideration. [29] Additional types of COI include academic conflicts (e.g. competition for funding) and conflicts related to clinical revenue streams (e.g., from performing an advanced diagnostic procedure that is under consideration for a recommendation).

#### Why are conflicted individuals not categorically excluded from guideline development?

The biases resulting from COI may be conscious or unconscious [43] and may influence choices made throughout the guideline development process, including conceptualization of the question, choice of comparisons, interpretation of the evidence and, in particular, drafting of the recommendations. [44] Under these circumstances why are conflicted individuals not categorically excluded from guideline development?

The most knowledgeable individuals can be conflicted because of their expertise in the area of interest. These “experts” may possess unique insight into appropriate healthcare questions to ask in guidelines. But because of their involvement in research they may have both financial and intellectual COIs whilst also possessing unique insight into aspects of the existing evidence such as study design and decisions made during conduct of studies. Such individuals may be difficult to replace because of the unique insights they provide. The aim (and challenge) should be to manage the potential COI appropriately ranging from informal consultation and exclusion from a group to participation without influencing recommendations. Approaches to doing this have been described in a recent policy of the American Thoracic Society [29]; an example is in a WHO guideline on Avian Influenza where un-conflicted methodologists prepared evidence summaries, chaired the guideline committee and wrote the first draft of the guideline. [45,46] A further implementation including a clearer separation of un-conflicted methodologists from the influence of potentially conflicted experts is currently undertaken by the executive committee of the American College of Chest Physicians Antithrombotic Guidelines. [47] In this latter approach un-conflicted methodologists lead the formulation of recommendations in collaboration with experts who may be conflicted to a degree that would not preclude them from participation.

### How should guideline developers deal with conflict of interest?

#### Declaration of COI

Participants in guideline development should ideally submit written disclosures of all potential interests that may cause a conflict of interest and disclosures should be made prior to being involved in an official capacity. The individual can declare in writing: all known current and past interests relevant to the subject and scope of the matter for a number years prior to the date of declaration (many organizations use a period of three years); and any conflicts of interest relevant to the subject and scope of the matter that are expected to occur in the near future. Disclosure can be made through standard forms or a uniform online process that could span across organizations. Updating as individualcircumstances warrant, and attest to its accuracy and currency is important. All participants (committee/group members and/or presenters) should be asked and reminded to consider their own conflicts and conflicts of others during discussions and decision making. Participants should abstain from discussion and voting if they or a sizable proportion of the other participants identify a COI. The chair of the group should be free of COI.

#### Review and management of COI

Those choosing participants for guideline development should review disclosures before deciding on participants, and consider excluding participants if there is a non-resolvable conflict of interest. The procedures (including step by step review and management) is best clearly described and part of a policy. This policy should involve independent review of COI and decision making by committees. Ideally guideline developers will provide group members with examples of COI management from anonymous cases that describe appropriate methods of COI management.

### Disclosure of COI to other group members

Once the members of a guideline group have been assembled, COI of members should be identified and discussed before beginning deliberations. Individual participants (including project chairs and group members) should label where COI bear on specific recommendations.

### Recusal or excusal from certain decisions or recommendations when appropriate

If groups involve members with (limited) COI, Chairs and group members on a guideline group should ensure that committees are reminded of the specific COI before discussion of individual conclusions or recommendations on which those COI bear. This will allow recusal from recommendations of those with important COI. Group chairs can play an active role and excuse group members from discussions or decision-making on particular recommendations.

### Procedures for handling disputes in COI resolution

An organization should develop and oversee the procedures and instruments used to disclose, review and resolve COI, and should advise and assist chairs and organizers where necessary. In instances where determination of COI and actions taken to resolve COI are disputed, procedures should be in place to address the matter.

### Summary

In this first paper we have described target audiences for guidelines, the criteria for choosing topics for guideline development (including acknowledging the current debate about co-morbidities), group composition and group processes and the important procedural issue of managing conflicts of interest. In the next paper we move on to discuss issues of identifying and synthesizing evidence: deciding what type of evidence and outcomes to include in guidelines; integrating values into a guideline; incorporating economic considerations; synthesis, grading, presentation of evidence; and moving from evidence to recommendations.

## Competing interests

MPE is Editor in Chief of Implementation Science; Jeremy Grimshaw is an Editorial Board member. All decisions on this paper were made by another editor. The authors have all been involved in guideline development for a range of different organizations.

## Authors’ contributions

All authors contributed to the writing of this article and approved the final draft.
